# 1-(4-Chloro­phen­yl)-1*H*-1,2,3,4-tetra­zole

**DOI:** 10.1107/S1600536812014353

**Published:** 2012-04-13

**Authors:** Jong Tae Kim, D. Gayathri, Vivek K. Gupta, Rajni Kant, Yeon Tae Jeong

**Affiliations:** a Department of Image Science and Engineering, Pukyong National University, Busan 608 739, Republic of Korea; b Department of Physics, Dr. M.G.R. Educational and Research Institute, Dr. M.G.R. University, Maduravoyal, Chennai 600 095, India; c X-ray Crystallography Laboratory, Post Graduate Department of Physics & Electronics, University of Jammu, Jammu Tawi 180 006, India

## Abstract

There are two independent mol­ecules in the asymmetric unit of the title compound, C_7_H_5_ClN_4_, in which the tetra­zole and benzene rings are twisted by dihedral angles of 12.9 (1) and 39.8 (1)°. In the crystal, the independent mol­ecules are connected into a tetra­mer by C—H⋯N hydrogen bonds, generating an *R*_4_^4^(12) graph-set motif.

## Related literature

For applications of tetra­zoles in medicinal and synthetic chemistry, see: Butler (1996 ▶). For related structures, see: Baek *et al.* (2012 ▶); Matsunaga *et al.* (1999 ▶); Lyakhov *et al.* (2000 ▶, 2001 ▶). For the synthesis, see: Aridoss & Laali (2011 ▶).
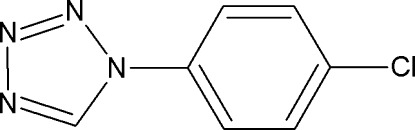


## Experimental

### 

#### Crystal data

C_7_H_5_ClN_4_
*M* *_r_* = 180.60
Monoclinic, 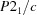

*a* = 3.8626 (2) Å
*b* = 27.9946 (10) Å
*c* = 14.4943 (5) Å
β = 95.640 (3)°
*V* = 1559.71 (11) Å^3^
*Z* = 8
Mo *K*α radiation
μ = 0.43 mm^−1^
*T* = 293 K
0.3 × 0.2 × 0.2 mm

#### Data collection

Oxford Diffraction Xcalibur Sapphire3 diffractometer
Absorption correction: multi-scan (*CrysAlis PRO*; Oxford Diffraction, 2010 ▶) *T*_min_ = 0.795, *T*_max_ = 0.917
16702 measured reflections
3366 independent reflections
2610 reflections with *I* > 2σ(*I*)
*R*_int_ = 0.038

#### Refinement

*R*[*F* ^2^ > 2σ(*F* ^2^)] = 0.049
*wR*(*F* ^2^) = 0.101
*S* = 1.07
3366 reflections
217 parameters
H-atom parameters constrained
Δρ_max_ = 0.18 e Å^−3^
Δρ_min_ = −0.22 e Å^−3^

### 

Data collection: *CrysAlis PRO* (Oxford Diffraction, 2010 ▶); cell refinement: *CrysAlis PRO*; data reduction: *CrysAlis PRO*; program(s) used to solve structure: *SHELXS97* (Sheldrick, 2008 ▶); program(s) used to refine structure: *SHELXL97* (Sheldrick, 2008 ▶); molecular graphics: *PLATON* (Spek, 2009 ▶); software used to prepare material for publication: *PLATON*.

## Supplementary Material

Crystal structure: contains datablock(s) I, global. DOI: 10.1107/S1600536812014353/is5104sup1.cif

Structure factors: contains datablock(s) I. DOI: 10.1107/S1600536812014353/is5104Isup2.hkl

Supplementary material file. DOI: 10.1107/S1600536812014353/is5104Isup3.cml

Additional supplementary materials:  crystallographic information; 3D view; checkCIF report

## Figures and Tables

**Table 1:** Hydrogen-bond geometry (Å, °)

*D*—H⋯*A*	*D*—H	H⋯*A*	*D*⋯*A*	*D*—H⋯*A*
C1*A*—H1*A*⋯N1*B*^i^	0.93	2.54	3.454 (3)	167
C1*B*—H1*B*⋯N1*A*^ii^	0.93	2.50	3.406 (3)	163

Symmetry codes: (i) 
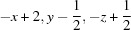
; (ii) 
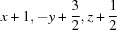
.

**Fig. 1.**
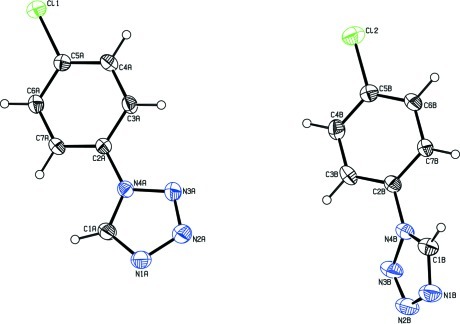
The asymmetric unit of the title compound, showing 30% probability displacement ellipsoids.

**Fig. 2.**
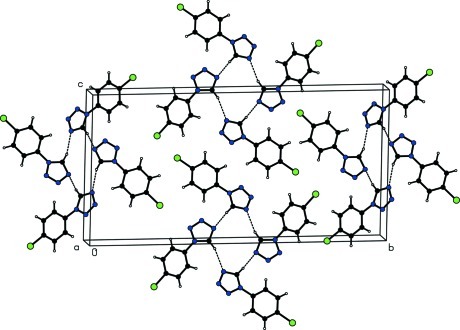
A molecular packing diagram of the title compound, showing intermolecular interactions.

## supplementary crystallographic information

### Comment

Heterocycles in general received much importance in recent years. Tetrazoles represent a unique class of heterocyclic compounds that exhibit a broad range of application both in medicinal and synthetic chemistry (Butler, 1996).

The title compound crystallizes in a monoclinic crystal system with two independent molecules in the asymmetric unit. Bond lengths and angles are comparable with the similar crystal structures (Baek *et al.*, 2012; Lyakhov *et al.*, 2000, 2001; Matsunaga *et al.*, 1999). The tetrazole and benzene rings are planar but not coplanar with the dihedral angle being 12.9 (1) and 39.8 (1)°, respectively, for molecules A and B. Torsion angles C1A—N4A—C2A—C3A [165.7 (2)°] and C1B—N4B—C2B—C3B [-138.5 (3)°] indicate for the difference in the dihedral angle between the two rings in molecules A and B. The chlorine atoms in molecules A and B deviate 0.021 (3) and 0.009 (3) Å, respectively, from the benzene plane. The crystal packing is stabilized by C—H···N intermolecular interactions (Table 1), wherein atom C1 acts as donor to N1 generating an *R*_4_^4^(12) graph-set motif.

### Experimental

The title compound was synthesized from the known procedure (Aridoss & Laali, 2011). Fine white diffraction quality crystals were obtained by slow evaporation of its ethanol solution.

### Refinement

All H-atoms were refined using a riding model, with C—H = 0.93 Å, and with *U*_iso_(H) = 1.2*U*_eq_(C).

### Crystal data

**Table:**

C_7_H_5_ClN_4_	*F*(000) = 736
*M**_r_* = 180.60	*D*_x_ = 1.538 Mg m^−^^3^
Monoclinic, *P*2_1_/*c*	Mo *K*α radiation, λ = 0.71073 Å
Hall symbol: -P 2ybc	Cell parameters from 7146 reflections
*a* = 3.8626 (2) Å	θ = 3.6–29.0°
*b* = 27.9946 (10) Å	µ = 0.43 mm^−^^1^
*c* = 14.4943 (5) Å	*T* = 293 K
β = 95.640 (3)°	Block, white
*V* = 1559.71 (11) Å^3^	0.3 × 0.2 × 0.2 mm
*Z* = 8	

### Data collection

**Table:**

Oxford Diffraction Xcalibur Sapphire3 diffractometer	3366 independent reflections
Radiation source: fine-focus sealed tube	2610 reflections with *I* > 2σ(*I*)
Graphite monochromator	*R*_int_ = 0.038
Detector resolution: 16.1049 pixels mm^-1^	θ_max_ = 27.0°, θ_min_ = 3.6°
ω scans	*h* = −4→4
Absorption correction: multi-scan (*CrysAlis PRO*; Oxford Diffraction, 2010)	*k* = −35→35
*T*_min_ = 0.795, *T*_max_ = 0.917	*l* = −18→18
16702 measured reflections	

### Refinement

**Table:**

Refinement on *F*^2^	Primary atom site location: structure-invariant direct methods
Least-squares matrix: full	Secondary atom site location: difference Fourier map
*R*[*F*^2^ > 2σ(*F*^2^)] = 0.049	Hydrogen site location: inferred from neighbouring sites
*wR*(*F*^2^) = 0.101	H-atom parameters constrained
*S* = 1.07	*w* = 1/[σ^2^(*F*_o_^2^) + (0.030*P*)^2^ + 0.7614*P*] where *P* = (*F*_o_^2^ + 2*F*_c_^2^)/3
3366 reflections	(Δ/σ)_max_ = 0.001
217 parameters	Δρ_max_ = 0.18 e Å^−^^3^
0 restraints	Δρ_min_ = −0.22 e Å^−^^3^

### Special details

**Table:**

Geometry. All e.s.d.'s (except the e.s.d. in the dihedral angle between two l.s. planes) are estimated using the full covariance matrix. The cell e.s.d.'s are taken into account individually in the estimation of e.s.d.'s in distances, angles and torsion angles; correlations between e.s.d.'s in cell parameters are only used when they are defined by crystal symmetry. An approximate (isotropic) treatment of cell e.s.d.'s is used for estimating e.s.d.'s involving l.s. planes.
Refinement. Refinement of *F*^2^ against ALL reflections. The weighted *R*-factor *wR* and goodness of fit *S* are based on *F*^2^, conventional *R*-factors *R* are based on *F*, with *F* set to zero for negative *F*^2^. The threshold expression of *F*^2^ > σ(*F*^2^) is used only for calculating *R*-factors(gt) *etc*. and is not relevant to the choice of reflections for refinement. *R*-factors based on *F*^2^ are statistically about twice as large as those based on *F*, and *R*- factors based on ALL data will be even larger.

### Fractional atomic coordinates and isotropic or equivalent isotropic displacement parameters (Å^2^)

**Table:**

	*x*	*y*	*z*	*U*_iso_*/*U*_eq_	
C1A	0.6813 (6)	0.49499 (9)	0.21942 (16)	0.0471 (6)	
H1A	0.7806	0.4769	0.1750	0.057*	
C2A	0.8047 (5)	0.44145 (7)	0.35903 (15)	0.0334 (5)	
C3A	0.8223 (6)	0.44243 (8)	0.45455 (15)	0.0406 (5)	
H3A	0.7443	0.4691	0.4848	0.049*	
C4A	0.9559 (6)	0.40373 (8)	0.50482 (16)	0.0420 (5)	
H4A	0.9670	0.4040	0.5692	0.050*	
C5A	1.0728 (5)	0.36472 (8)	0.45945 (15)	0.0373 (5)	
C6A	1.0536 (6)	0.36342 (8)	0.36386 (16)	0.0434 (5)	
H6A	1.1318	0.3368	0.3338	0.052*	
C7A	0.9169 (6)	0.40214 (8)	0.31318 (15)	0.0424 (5)	
H7A	0.9010	0.4016	0.2487	0.051*	
N1A	0.5321 (6)	0.53656 (7)	0.20473 (15)	0.0525 (5)	
N2A	0.4223 (6)	0.54976 (7)	0.28770 (15)	0.0561 (6)	
N3A	0.5037 (6)	0.51752 (7)	0.34968 (14)	0.0523 (5)	
N4A	0.6693 (4)	0.48236 (6)	0.30800 (12)	0.0368 (4)	
Cl1	1.24583 (17)	0.31657 (2)	0.52393 (4)	0.05170 (18)	
C1B	0.9963 (7)	0.92383 (9)	0.51988 (17)	0.0507 (6)	
H1B	1.1251	0.9410	0.5663	0.061*	
C2B	1.0545 (6)	0.84159 (8)	0.58921 (15)	0.0378 (5)	
C3B	1.1628 (6)	0.79776 (9)	0.56058 (17)	0.0481 (6)	
H3B	1.1582	0.7909	0.4977	0.058*	
C4B	1.2783 (6)	0.76411 (9)	0.62583 (18)	0.0493 (6)	
H4B	1.3517	0.7343	0.6073	0.059*	
C5B	1.2846 (6)	0.77494 (8)	0.71897 (16)	0.0410 (5)	
C6B	1.1770 (6)	0.81872 (8)	0.74729 (16)	0.0465 (6)	
H6B	1.1841	0.8257	0.8102	0.056*	
C7B	1.0581 (6)	0.85244 (8)	0.68223 (16)	0.0429 (5)	
H7B	0.9814	0.8821	0.7008	0.052*	
N1B	0.8456 (6)	0.94204 (9)	0.44305 (16)	0.0615 (6)	
N2B	0.6892 (6)	0.90440 (10)	0.39623 (15)	0.0644 (6)	
N3B	0.7429 (6)	0.86506 (9)	0.44239 (15)	0.0578 (6)	
N4B	0.9373 (5)	0.87688 (7)	0.52159 (12)	0.0418 (5)	
Cl2	1.43462 (19)	0.73209 (2)	0.80028 (5)	0.0613 (2)	

### Atomic displacement parameters (Å^2^)

**Table:**

	*U*^11^	*U*^22^	*U*^33^	*U*^12^	*U*^13^	*U*^23^
C1A	0.0595 (15)	0.0454 (14)	0.0359 (13)	0.0024 (12)	0.0023 (11)	−0.0014 (11)
C2A	0.0320 (11)	0.0354 (11)	0.0327 (12)	−0.0031 (9)	0.0031 (8)	−0.0017 (9)
C3A	0.0485 (14)	0.0385 (12)	0.0355 (13)	0.0007 (10)	0.0082 (10)	−0.0075 (10)
C4A	0.0502 (14)	0.0453 (13)	0.0305 (12)	−0.0030 (11)	0.0048 (10)	−0.0025 (10)
C5A	0.0346 (11)	0.0389 (12)	0.0384 (12)	−0.0026 (9)	0.0028 (9)	0.0003 (10)
C6A	0.0500 (14)	0.0402 (12)	0.0406 (13)	0.0049 (10)	0.0080 (10)	−0.0065 (11)
C7A	0.0512 (14)	0.0474 (13)	0.0290 (12)	0.0029 (11)	0.0059 (10)	−0.0050 (10)
N1A	0.0636 (14)	0.0467 (12)	0.0455 (13)	0.0026 (10)	−0.0029 (10)	0.0016 (10)
N2A	0.0685 (15)	0.0482 (12)	0.0507 (14)	0.0092 (11)	0.0021 (11)	0.0003 (11)
N3A	0.0667 (14)	0.0457 (12)	0.0454 (13)	0.0135 (10)	0.0098 (10)	−0.0020 (10)
N4A	0.0395 (10)	0.0353 (10)	0.0354 (10)	−0.0021 (8)	0.0030 (8)	−0.0035 (8)
Cl1	0.0571 (4)	0.0493 (3)	0.0477 (4)	0.0085 (3)	0.0000 (3)	0.0054 (3)
C1B	0.0543 (15)	0.0572 (16)	0.0404 (14)	−0.0027 (12)	0.0032 (11)	0.0054 (12)
C2B	0.0355 (11)	0.0440 (12)	0.0341 (12)	−0.0057 (10)	0.0050 (9)	−0.0016 (10)
C3B	0.0568 (15)	0.0546 (15)	0.0341 (13)	−0.0037 (12)	0.0101 (11)	−0.0107 (12)
C4B	0.0551 (15)	0.0434 (13)	0.0504 (16)	0.0031 (11)	0.0100 (12)	−0.0081 (12)
C5B	0.0358 (12)	0.0426 (12)	0.0442 (14)	−0.0030 (10)	0.0027 (10)	0.0035 (11)
C6B	0.0565 (15)	0.0508 (14)	0.0320 (12)	0.0020 (12)	0.0033 (10)	−0.0053 (11)
C7B	0.0506 (14)	0.0416 (12)	0.0369 (13)	0.0048 (11)	0.0059 (10)	−0.0060 (10)
N1B	0.0652 (15)	0.0721 (15)	0.0469 (14)	0.0012 (12)	0.0039 (11)	0.0161 (12)
N2B	0.0654 (15)	0.0881 (18)	0.0385 (12)	0.0015 (14)	−0.0006 (11)	0.0108 (13)
N3B	0.0596 (14)	0.0760 (16)	0.0356 (12)	−0.0056 (12)	−0.0065 (10)	−0.0005 (11)
N4B	0.0410 (11)	0.0525 (12)	0.0316 (11)	−0.0038 (9)	0.0027 (8)	0.0008 (9)
Cl2	0.0659 (4)	0.0552 (4)	0.0607 (4)	0.0056 (3)	−0.0037 (3)	0.0109 (3)

### Geometric parameters (Å, º)

**Table:**

C1A—N1A	1.307 (3)	C1B—N1B	1.308 (3)
C1A—N4A	1.337 (3)	C1B—N4B	1.335 (3)
C1A—H1A	0.9300	C1B—H1B	0.9300
C2A—C7A	1.377 (3)	C2B—C3B	1.374 (3)
C2A—C3A	1.380 (3)	C2B—C7B	1.381 (3)
C2A—N4A	1.434 (3)	C2B—N4B	1.433 (3)
C3A—C4A	1.378 (3)	C3B—C4B	1.378 (3)
C3A—H3A	0.9300	C3B—H3B	0.9300
C4A—C5A	1.374 (3)	C4B—C5B	1.381 (3)
C4A—H4A	0.9300	C4B—H4B	0.9300
C5A—C6A	1.381 (3)	C5B—C6B	1.370 (3)
C5A—Cl1	1.736 (2)	C5B—Cl2	1.741 (2)
C6A—C7A	1.385 (3)	C6B—C7B	1.381 (3)
C6A—H6A	0.9300	C6B—H6B	0.9300
C7A—H7A	0.9300	C7B—H7B	0.9300
N1A—N2A	1.365 (3)	N1B—N2B	1.362 (3)
N2A—N3A	1.290 (3)	N2B—N3B	1.295 (3)
N3A—N4A	1.349 (2)	N3B—N4B	1.350 (3)
			
N1A—C1A—N4A	109.6 (2)	N1B—C1B—N4B	109.7 (2)
N1A—C1A—H1A	125.2	N1B—C1B—H1B	125.2
N4A—C1A—H1A	125.2	N4B—C1B—H1B	125.2
C7A—C2A—C3A	120.9 (2)	C3B—C2B—C7B	121.2 (2)
C7A—C2A—N4A	120.34 (19)	C3B—C2B—N4B	119.6 (2)
C3A—C2A—N4A	118.77 (19)	C7B—C2B—N4B	119.2 (2)
C4A—C3A—C2A	119.6 (2)	C2B—C3B—C4B	119.4 (2)
C4A—C3A—H3A	120.2	C2B—C3B—H3B	120.3
C2A—C3A—H3A	120.2	C4B—C3B—H3B	120.3
C5A—C4A—C3A	119.7 (2)	C3B—C4B—C5B	119.6 (2)
C5A—C4A—H4A	120.1	C3B—C4B—H4B	120.2
C3A—C4A—H4A	120.1	C5B—C4B—H4B	120.2
C4A—C5A—C6A	120.9 (2)	C6B—C5B—C4B	120.8 (2)
C4A—C5A—Cl1	119.12 (17)	C6B—C5B—Cl2	120.29 (19)
C6A—C5A—Cl1	119.99 (17)	C4B—C5B—Cl2	118.89 (18)
C5A—C6A—C7A	119.5 (2)	C5B—C6B—C7B	119.8 (2)
C5A—C6A—H6A	120.3	C5B—C6B—H6B	120.1
C7A—C6A—H6A	120.3	C7B—C6B—H6B	120.1
C2A—C7A—C6A	119.4 (2)	C6B—C7B—C2B	119.2 (2)
C2A—C7A—H7A	120.3	C6B—C7B—H7B	120.4
C6A—C7A—H7A	120.3	C2B—C7B—H7B	120.4
C1A—N1A—N2A	105.5 (2)	C1B—N1B—N2B	105.2 (2)
N3A—N2A—N1A	110.40 (19)	N3B—N2B—N1B	111.1 (2)
N2A—N3A—N4A	107.00 (19)	N2B—N3B—N4B	106.2 (2)
C1A—N4A—N3A	107.45 (18)	C1B—N4B—N3B	107.9 (2)
C1A—N4A—C2A	131.41 (19)	C1B—N4B—C2B	130.4 (2)
N3A—N4A—C2A	121.11 (18)	N3B—N4B—C2B	121.6 (2)
			
C7A—C2A—C3A—C4A	0.4 (3)	C7B—C2B—C3B—C4B	−0.3 (4)
N4A—C2A—C3A—C4A	−179.11 (19)	N4B—C2B—C3B—C4B	179.4 (2)
C2A—C3A—C4A—C5A	0.5 (3)	C2B—C3B—C4B—C5B	−0.2 (4)
C3A—C4A—C5A—C6A	−0.9 (3)	C3B—C4B—C5B—C6B	0.0 (4)
C3A—C4A—C5A—Cl1	179.15 (17)	C3B—C4B—C5B—Cl2	−179.50 (18)
C4A—C5A—C6A—C7A	0.5 (3)	C4B—C5B—C6B—C7B	0.5 (4)
Cl1—C5A—C6A—C7A	−179.63 (17)	Cl2—C5B—C6B—C7B	−179.93 (18)
C3A—C2A—C7A—C6A	−0.9 (3)	C5B—C6B—C7B—C2B	−0.9 (4)
N4A—C2A—C7A—C6A	178.63 (19)	C3B—C2B—C7B—C6B	0.8 (3)
C5A—C6A—C7A—C2A	0.5 (3)	N4B—C2B—C7B—C6B	−178.8 (2)
N4A—C1A—N1A—N2A	−0.4 (3)	N4B—C1B—N1B—N2B	−0.1 (3)
C1A—N1A—N2A—N3A	0.3 (3)	C1B—N1B—N2B—N3B	−0.1 (3)
N1A—N2A—N3A—N4A	0.0 (3)	N1B—N2B—N3B—N4B	0.3 (3)
N1A—C1A—N4A—N3A	0.4 (3)	N1B—C1B—N4B—N3B	0.3 (3)
N1A—C1A—N4A—C2A	−177.6 (2)	N1B—C1B—N4B—C2B	178.2 (2)
N2A—N3A—N4A—C1A	−0.2 (3)	N2B—N3B—N4B—C1B	−0.4 (3)
N2A—N3A—N4A—C2A	178.05 (19)	N2B—N3B—N4B—C2B	−178.4 (2)
C7A—C2A—N4A—C1A	−13.8 (3)	C3B—C2B—N4B—C1B	−138.5 (3)
C3A—C2A—N4A—C1A	165.7 (2)	C7B—C2B—N4B—C1B	41.1 (3)
C7A—C2A—N4A—N3A	168.4 (2)	C3B—C2B—N4B—N3B	39.1 (3)
C3A—C2A—N4A—N3A	−12.0 (3)	C7B—C2B—N4B—N3B	−141.3 (2)

### Hydrogen-bond geometry (Å, º)

**Table:**

*D*—H···*A*	*D*—H	H···*A*	*D*···*A*	*D*—H···*A*
C1*A*—H1*A*···N1*B*^i^	0.93	2.54	3.454 (3)	167
C1*B*—H1*B*···N1*A*^ii^	0.93	2.50	3.406 (3)	163

Symmetry codes: (i) −*x*+2, *y*−1/2, −*z*+1/2; (ii) *x*+1, −*y*+3/2, *z*+1/2.
